# Moderating Effect of Depression on Glycemic Control in an eHealth Intervention Among Black Youth With Type 1 Diabetes: Findings From a Multicenter Randomized Controlled Trial

**DOI:** 10.2196/55165

**Published:** 2024-04-09

**Authors:** Deborah Ellis, April Idalski Carcone, Thomas Templin, Meredyth Evans, Jill Weissberg-Benchell, Colleen Buggs-Saxton, Claudia Boucher-Berry, Jennifer L Miller, Tina Drossos, M Bassem Dekelbab

**Affiliations:** 1 Department of Family Medicine and Public Health Sciences Wayne State University School of Medicine Detroit, MI United States; 2 College of Nursing Wayne State University Detroit, MI United States; 3 Pritzker Department of Psychiatry and Behavioral Health Ann and Robert H Lurie Children’s Hospital Chicago, IL United States; 4 Department of Psychiatry and Behavioral Sciences Northwestern Feinberg School of Medicine Chicago, IL United States; 5 Department of Pediatrics Wayne State University School of Medicine Detroit, MI United States; 6 Department of Pediatrics University of Illinois School of Medicine at Chicago Chicago, IL United States; 7 Department of Pediatrics Northwestern Feinberg School of Medicine Chicago, IL United States; 8 Department of Psychiatry and Behavioral Neurosciences University of Chicago Pritzker School of Medicine Chicago, IL United States; 9 Corewell Health Royal Oak, MI United States

**Keywords:** adolescents, black, depression, eHealth, family intervention, randomized clinical trial, randomized controlled trial, T1D, type 1 diabetes

## Abstract

**Background:**

Black adolescents with type 1 diabetes (T1D) are at increased risk for suboptimal diabetes health outcomes; however, evidence-based interventions for this population are lacking. Depression affects a high percentage of youth with T1D and increases the likelihood of health problems associated with diabetes.

**Objective:**

Our aim was to test whether baseline levels of depression moderate the effects of a brief eHealth parenting intervention delivered to caregivers of young Black adolescents with T1D on youths’ glycemic control.

**Methods:**

We conducted a multicenter randomized controlled trial at 7 pediatric diabetes clinics located in 2 large US cities. Participants (N=149) were allocated to either the intervention group or a standard medical care control group. Up to 3 intervention sessions were delivered on a tablet computer during diabetes clinic visits over a 12-month period.

**Results:**

In a linear mixed effects regression model, planned contrasts did not show significant reductions in hemoglobin A_1c_ (HbA_1c_) for intervention adolescents compared to controls. However, adolescents with higher baseline levels of depressive symptoms who received the intervention had significantly greater improvements in HbA_1c_ levels at 6-month follow-up (0.94%; *P*=.01) and 18-month follow-up (1.42%; *P*=.002) than those with lower levels of depression. Within the intervention group, adolescents had a statistically significant reduction in HbA_1c_ levels from baseline at 6-month and 18-month follow-up.

**Conclusions:**

A brief, culturally tailored eHealth parenting intervention improved health outcomes among Black adolescents with T1D and depressive symptoms.

**Trial Registration:**

ClinicalTrials.gov NCT03168867; https://clinicaltrials.gov/study/NCT03168867

## Introduction

Adolescence is a period of risk for youth with type 1 diabetes (T1D), as the transition to independent diabetes management is challenging for families to navigate [1], affecting glycemic control [2]. Black adolescents with T1D are at even higher risk for diabetes-related health disparities, such as elevated blood glucose levels [3], hospital admissions [4], and diabetes distress [5]. Given the critical protective role played by families in the health of adolescents with T1D, a variety of family-based interventions have been developed. Such interventions have used multiple strategies to target the family process related to youth diabetes health, such as improving diabetes-related family communication and reducing conflict [6]. However, despite the extensive literature documenting health disparities, few randomized controlled trials have included adequate samples of Black adolescents with T1D [7]. Almost no clinical trials have tested interventions designed and tailored for Black adolescents and their families [8,9].

eHealth interventions have shown promising effects for a number of health conditions, including T1D [10], and circumvent many of the barriers that prevent successful behavioral interventions from being adopted [11]. Behavioral health services are also limited in many pediatric diabetes care settings by the lack of trained mental health specialists [12], despite widespread acknowledgment of their value [13]. Furthermore, as family-centered care approaches have been shown to improve health outcomes in youth with T1D, there have been a growing number of calls to leverage technological advancements to promote the use of family-centered care through internet-based or other similar eHealth tools and interventions [14]. As regular attendance at diabetes clinics is part of the recommendations for the care of adolescents with T1D [15], such visits may provide a natural opportunity to deliver such eHealth interventions.

In collaboration with Black adolescents with T1D and their caregivers, we previously developed and tested the feasibility of a brief, culturally tailored eHealth intervention (The 3Ms) [16,17], aimed at increasing a critical protective parenting practice: daily parental monitoring of adolescent diabetes care [18-20]. While parents often reduce involvement in diabetes care during adolescence, decreased involvement is associated with suboptimal glycemic control [21,22]. Therefore, the intervention was developed for primary caregivers of young Black adolescents with T1D transitioning to independent self-care to decrease parental disengagement from diabetes management during this high-risk developmental period.

Depression, including symptoms of hopelessness and helplessness, affects approximately 20% of youth with T1D [23]. Multiple studies have shown that depression is a significant predictor of health outcomes in youth with T1D, as it may affect health through either suboptimal self-management [24] or physiological mechanisms such as metabolic abnormalities and systemic inflammation [25]. Cross-sectional and longitudinal studies have shown that youth with T1D and depression are also more likely to report family conflict and low levels of parental involvement in diabetes care [26,27]. Such findings suggest that elevated depressive symptoms may identify youth who are more likely to be treatment responders in behavioral studies aimed at increasing family support for diabetes management [28]. In order to determine how to most effectively tailor treatments and develop the best decision rules for choosing between treatment alternatives, it is crucial for moderator variables to be identified that predict for whom a particular intervention is most likely to succeed. While there is limited information on such moderator variables from previous trials of health behavior change interventions for adolescents with T1D, a clinical trial testing a web-based diabetes coping intervention found that adolescents with higher levels of depression at baseline had more improvements in quality of life at the conclusion of the study [29]. Other clinical trials testing health behavior change interventions for Black families have likewise shown that the baseline level of depression in adolescents is related to treatment response [30].

The purpose of this study was to investigate the effects of depression in adolescents as a potential moderator of the efficacy of The 3Ms to improve glycemic control in a randomized controlled trial.

## Methods

### Ethical Considerations

This study was approved by the institutional review board of the first author’s university (IRB# 015117B3E) using a single institutional review board agreement covering all participating institutions. The primary caregiver and adolescent provided informed consent and assent to participate. Participants were provided with US $50 at each study visit to compensate them for their participation. The trial was registered at ClinicalTrials.gov (NCT03168867).

### Procedures

Adolescent participants and their primary caregivers were recruited from 3 pediatric diabetes clinics located in the greater metropolitan Detroit area and 4 in the Chicago area. The study took place from 2017 to 2021. Eligible adolescents had to be aged between 10 years, 0 months, and 14 years, 11 months, diagnosed with T1D for at least 6 months, self-identify as Black, and be residing with a caregiver who was willing to participate in the study. Study exclusion criteria were psychiatric diagnoses, such as suicidal ideation or psychosis, cognitive impairments that limited the ability to complete study measures, not being able to speak in English, or having an additional medical diagnosis leading to atypical diabetes management.

The data regarding study eligibility (based on adolescents’ age, race, and medical diagnosis) were obtained from the electronic medical records of the participating diabetes clinics, along with their contact information. Families were first sent an introductory letter describing the study. Subsequently, study research staff contacted the adolescent’s primary caregiver by phone or at a clinic visit to provide more information and screen interested families for additional eligibility criteria.

**Figure 1:**
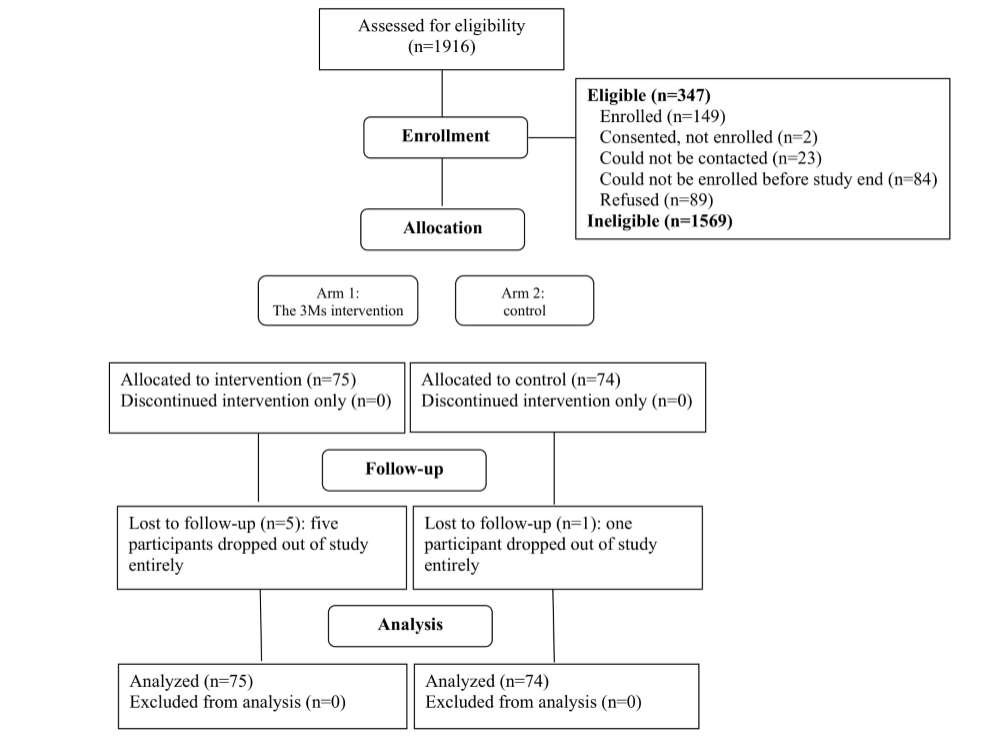
The CONSORT (Consolidated Standards of Reporting Trials) flow diagram.

Of the 1916 families screened for participation, 1569 were ineligible, and 23 could not be contacted. Of the remaining 324, a total of 89 (27.5%) declined to participate, citing lack of interest or time. An additional 86 (26.5%) expressed an interest in the study but did not enroll before the closure of recruitment. A total of 149 families were enrolled (89 from Detroit clinics and 60 from Chicago clinics), of whom 75 were assigned to The 3Ms and 74 to standard care. A total of 5 of The 3Ms families and 1 of the standard care families dropped out of the study and did not complete follow-up data collection. The overall study retention rate was 96% (143/149). Enrollment and flow through the study are shown in Figure 1.

The study was a multicenter controlled trial using a randomized, controlled, parallel arm design. Participants were allocated to either The 3Ms plus standard medical care or standard medical care control in a 1:1 ratio using block randomization within 14 strata defined by the 7 sites and hemoglobin A_1c_ (HbA_1c_) level (most recent HbA_1c_ <9.5% vs ≥9.5%) after baseline data collection. The allocation sequence was generated by the study statistician using randomization software. Assignment to condition was completed by study research staff immediately after baseline by opening a sequentially numbered, sealed envelope with the allocation.

This study was designed as an effectiveness trial to test the effects of The 3Ms under “real-world” conditions. Caregivers who were randomized to receive The 3Ms completed between 1 and 3 intervention sessions, depending on the number of diabetes clinic visits attended by the family during the 12-month intervention window. A maximum dose of 3 sessions was chosen based upon routine practice in the care of youth with diabetes [15], as standards of care include quarterly visits to a diabetes specialty care center. The first intervention session was delivered after the baseline data collection to ensure all caregivers received at least 1 intervention session. The subsequent 2 sessions were completed during any clinic visit that occurred during the 12 months after baseline.

The planned study design called for follow-up data collection visits to be completed in the family home to minimize study attrition. T2 data collection visits were completed 6 months after baseline, T3 data collection visits were completed 13 months after baseline (1 month after the 12-month intervention window was complete), and T4 data collection visits were completed 18 months after baseline (6 months after intervention completion). However, the COVID-19 pandemic caused the study’s institutional review board to place restrictions on all face-to-face contact with trial participants in March of 2020, which precluded any subsequent in-home data collection. For follow-up data collections completed after this date, participants either had HbA_1c_ test kits dropped at their home or were mailed the test kit to complete and return. In both cases, study staff watched the adolescents complete the test during a videoconference call to ensure reliable collection of the specimen. Due to the difficulties associated with the completion of study follow-up visits during the pandemic, the planned study design, in which follow-up data were only collected within narrow study windows (±2 weeks from the planned visit date, or 30 days in total), were modified to obtain data whenever possible within 18 months after baseline. About 87.3% (124/142), 86.7% (118/136), and 87.4% (106/121) of data collection visits were within a 45-day window of the planned visit dates at T2, T3, and T4, respectively. Study staff were not blinded to the treatment conditions; however, the objective nature of the HbA_1c_ measure mitigated the risk of bias.

The 3Ms intervention was delivered using Computer Intervention Authoring Software, an internet-based, interactional software [31]. Session content was delivered by an interactional and emotive 3-dimensional narrator that reads, speaks aloud, reflects participant responses, and functions as an engaging guide throughout the intervention. This approach is particularly useful in populations such as those for whom the present intervention was designed, where challenges with health literacy could affect engagement with the eHealth intervention [32]. Caregivers used a tablet computer provided to them at the diabetes clinic visit by research staff to complete The 3Ms.

The early development process for The 3Ms intervention has been reported elsewhere [17], as have the results of pilot testing [16]. In brief, The 3Ms was based on the “Information-motivation-behavioral Skills” model of behavior change [33], which posits that health behavior change is driven by 3 critical components: “accurate information” about both risk behaviors and their replacement health behaviors (eg, benefits of daily parental monitoring), “motivation” to change behavior, and “behavioral skills and confidence” (eg, self-efficacy) necessary to perform the behavior. As The 3Ms was designed to be delivered during regular diabetes clinic visits, each session lasted approximately 15-20 minutes. To ensure the cultural relevance of The 3Ms for Black caregivers, the early intervention development process included input and review of intervention content and language from Black pediatric researchers and beta-testing by caregivers of Black adolescents with T1D.

**Figure 2:**
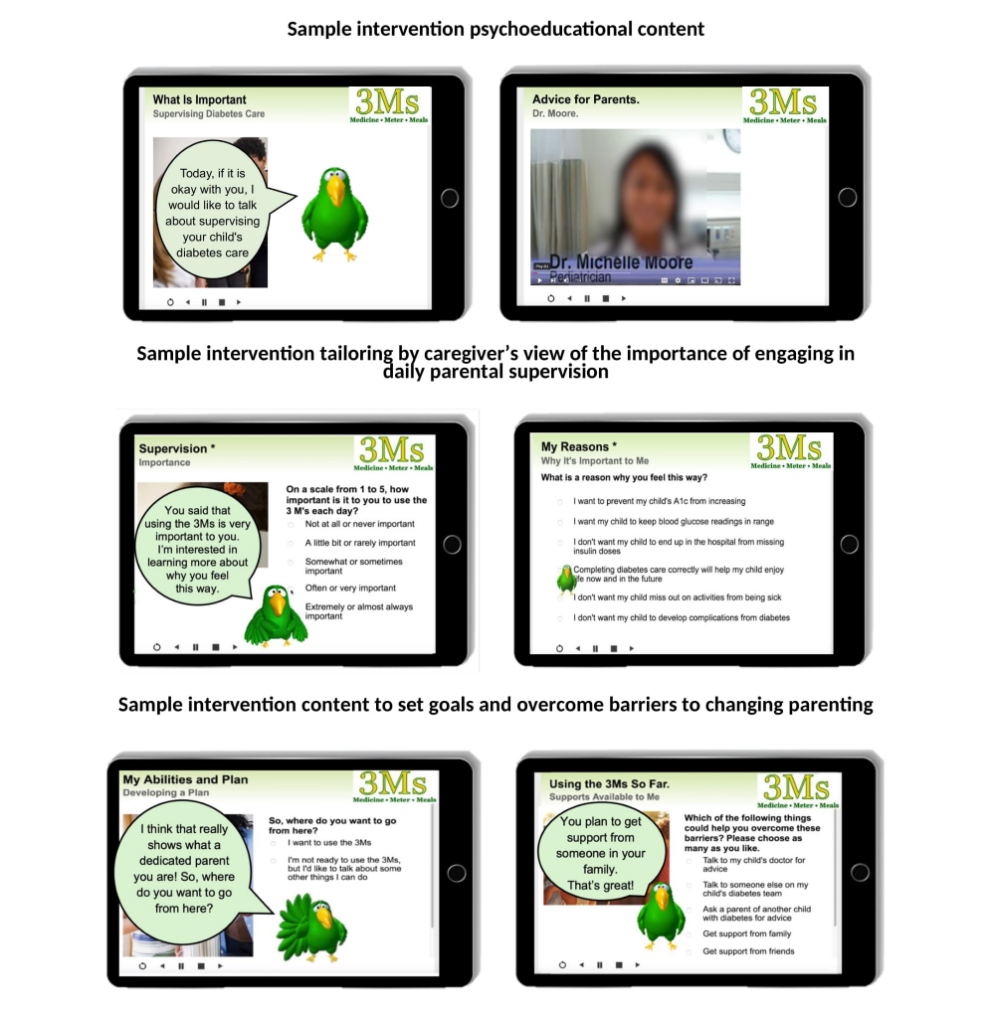
Sample intervention content for The 3Ms.

The intervention’s informational content encouraged parents to use 3 strategies for increasing parental supervision and monitoring of adolescent diabetes management. Called “The 3Ms,” the strategies were (1) watch your child give as many doses of insulin each day as possible (medicine), (2) check your child’s glucose monitor at least once a day (monitor), and (3) eat at least 1 meal each day with your child so carbohydrate counting can be assessed (meals). This informational content was delivered through psychoeducational video clips where a Black endocrinologist and a Black caregiver provided advice regarding these parenting behaviors and encouragement to use them. To increase caregivers’ motivation and self-efficacy to engage in daily supervision of adolescent diabetes management, the intervention used multiple strategies consistent with motivational interviewing [34], including evoking change talk and commitment language (ie, statements regarding desires, reasons, needs, and abilities to make behavior change) and eliciting the pros and cons of behavior change. Intervention content was tailored based on caregivers’ ratings of the importance of engaging in daily parental supervision and their ratings of self-efficacy for parental supervision. Tailoring also included the completion of different content in follow-up sessions based on caregiver appraisals of their success in completing daily parental supervision, as well as the completion of optional goal-setting activities at the end of each session (Figure 2 provides sample intervention content).

### Measures

HbA_1c_ level was used to evaluate glycemic control. Values were obtained during data collection visits using the Food and Drug Administration (FDA)–approved Accubase fingerstick capillary blood collection test kit. Due to the COVID-19 pandemic and higher than expected missed data collection visits, these data were also obtained from the clinic medical record for follow-up points if a clinic visit fell within ±30 days of data collection and data were otherwise missing. A total of 88.5% (485/548) of follow-up HbA_1c_ measurements were obtained using the Accubase test kit, and 11.5% (63/548) were obtained from the medical record. Previous studies have shown high comparability between samples collected using methods similar to those of the Accubase kit compared to venous samples [35].

A self-report questionnaire was used to obtain information from the adolescent’s primary caregiver on demographic variables. The adolescent’s medical chart was reviewed to obtain clinical information such as the duration of diabetes and insulin delivery method.

Adolescent depressive symptoms were measured at baseline using an adapted version of the 8-item Patient-Reported Outcome Measurement Information System Pediatric Short Form Depressive Symptoms (PROMIS-D; version 1.0) [36]. The self-report scale assesses mood, positive or negative affect, and views of self. Items were rated from 1 to 4, with higher scores reflecting more depression. The internal consistency of the measure in this study was high (α=.94). For the analyses, PROMIS-D was dichotomized at a score of 1 SD above the sample mean (<23 vs ≥23). This approach is similar to using a T-score of 60 or higher; PROMIS-D T-scores in this range indicate mood-related difficulties [37].

### Statistical Analyses

Analyses were conducted using a repeated-measures linear mixed effects (LME) regression model. The LME model included 3 fixed factors and 4 fixed covariates. The fixed factors were treatment group (The 3Ms vs control), data collection point (at baseline, 6 months, 13 months, and 18 months), and treatment moderator (PROMIS-D ≥23 vs <23). The 4 covariates were age, income, and 2 dummy codes for insulin delivery method. These covariates were selected from medical and demographic characteristics (Table 1). A correlation below the threshold value of *P*=.10 with either the treatment group variable or HbA_1c_ determined selection. The intercept and study site were random factors. The treatment effects were evaluated with change-from-baseline–planned comparisons in HbA_1c_ levels at 6-, 13-, and 18-month follow-up. Planned comparisons were statistically evaluated with a 2-sided *P*<.05 for significance. Moderation effects were investigated with post hoc simple effect tests.

**Table 1:** Demographic characteristics of adolescents and primary caregivers.

Variable			Total sample (N=149)			The 3Ms group (n=75)			The control group (n=74)			
Adolescent age (years), mean (SD)			13.4 (1.7)			13.1 (1.8)			13.7 (1.5)			
**Adolescents’ sex, n (%)**												
	Male	63 (42.3)			29 (38.7)			34 (45.9)				
	Female	86 (57.7)			46 (63.1)			40 (54.1)				
Duration of diabetes (years), mean (SD)			5.8 (3.9)			5.6 (3.9)			6.1 (3.8)			
**HbA_1c_, mean (SD)**												
	%	11.5 (2.7)			11.5 (2.7)			11.5 (2.8)				
	mmol/mol	102.1 (29.7)			102.3 (29.1)			102.0 (30.5)				
**Insulin delivery, n (%)**												
	Basal bolus injection	98 (65.8)			53 (70.7)			45 (60.8)				
	Basal bolus pump	41 (27.5)			17 (22.6)			24 (32.4)				
	Other	10 (6.7)			5 (6.7)			5 (6.8)				
Caregivers’ age (years), mean (SD)			42.4 (8.7)			42.3 (9.0)			42.5 (8.5)			
**Caregivers’ sex, n (%)**												
	Female	134 (89.9)			67 (89.3)			67 (90.5)				
	Male	15 (10.1)			8 (11.7)			7 (9.5)				
**Caregivers’ race, n (%)**												
	Black	139 (93.3)			72 (96.0)			67 (90.5)				
	Other	10 (6.7)			3 (4.0)			7 (9.5)				
Caregivers’ education (years), mean (SD)			13.4 (2.3)			13.2 (2.3)			13.6 (2.2)			
**Number of caregivers in the home, n (%)**												
	2			74 (49.6)			46 (61.3)			28 (37.8)		
	1	75 (51.4)			29 (39.7)			46 (62.2)				
Yearly family income (US $), mean (SD)			34,933 (27,076)			36,644 (26,511)			33,889 (27,961)			

Analyses were intent-to-treat, and all randomized cases were included. Of the 149 enrolled cases, 122 cases provided complete HbA_1c_ data across 18 months. Under the assumption that data are missing at random, the LME model used all available data to estimate model parameters. Explicit data imputation was not required.

## Results

Sample characteristics are presented in Table 1. The mean age was 13.4 (SD 1.7; range 10.1-15.9) years. The mean HbA_1c_ level expressed as a percentage was 11.5% (SD 2.7%; range 5.3%-18.2%) and that expressed as mmol/mol was 102.1 (SD 29.7; range 34.4-175.4) mmol/mol, suggesting that the sample’s glycemic control was outside of the recommended range, consistent with known disparities in glycemic outcomes for Black youth [3,4]. The majority of adolescents (108/149, 72.5%) were managed with injected insulin, while 27.5% (41/149) used insulin pumps. The mean yearly family income was US $34,933 (SD US $27,076; range US $5000-US $105,000), and the median was US $25,000 (IQR US $15,000-US $55,000), corresponding to approximately 95% of the US 2020 poverty line for a family of 4.

The mean HbA_1c_ level expressed as a percentage was 11.5% (SD 2.7%; range 5.3%-17.8%), and that expressed in mmol/mol was 102.3 (SD 29.1; range 34.4-171.1) mmol/mol in The 3Ms condition, and 11.5% (SD 2.8%; range 6.7%-18.2%) and 102.0 (SD 30.5, range 49.7-175.4) mmol/mol in the control condition, respectively, with no significant difference between groups. A total of 24.8% (37/149) of the youth in the sample fell at or above the PROMIS-D cutoff score of 23, suggesting they had elevated depressive symptoms. The number of The 3Ms sessions received was evenly distributed across the sample, with 36% (27/75), 36% (27/75), and 28% (21/75) of caregivers in The 3Ms group receiving 1, 2, and 3 sessions, respectively.

**Figure 3:**
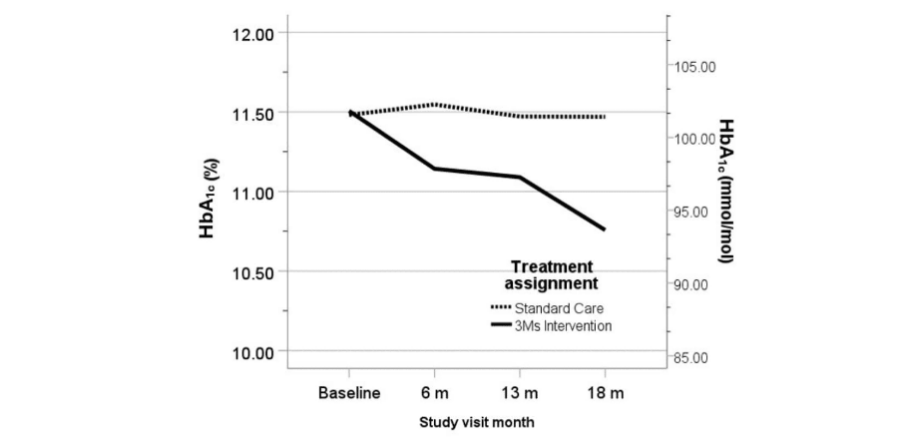
Hemoglobin A_1c_ (HbA_1c_) trajectories by intervention group from baseline to 18 months.

Adolescents assigned to The 3Ms had lower HbA_1c_ levels at each of the postbaseline assessments relative to the control group, with a reduction in HbA_1c_ relative to the control condition of 0.56% (5.99 mmol/mol) at 6-month follow-up (*P*=.10), 0.42% (4.50 mmol/mol) at 13-month (*P*=.28) follow-up, and 0.68% at 18-month follow-up (*P*=.09; Figure 3).

Planned group×time contrasts were not significant (Table 2 provides between-group differences). However, the change in HbA_1c_ within The 3Ms group was statistically significant and was also clinically significant (≥0.5%). Adolescents assigned to The 3Ms had a significant reduction in HbA_1c_ levels of 0.53% (5.70 mmol/mol) at 6-month follow-up (*P*=.02), and 0.83% (2.07 mmol/mol) at 18 months (*P*=.002; Table 2 provides changes from baseline). The change in HbA_1c_ levels from baseline within the control group was small at each time point (ie, less than 0.15%) and not significant.

**Table 2:** Changes in hemoglobin A_1c_ (HbA_1c_) levels at 6, 13, and 18 months after baseline. At baseline, N=149, with 74 in the control condition and 75 in the intervention group. Mean estimates and statistical tests used the linear mixed effect model with covariates held at their mean level with conventionally injected insulin=.07, insulin pump=.27, adolescent age=13.38 years, family income=US $35,731, and using all available data.

Study visit	Metric	Changes from baseline									Between-group differences^a^							Frequency, n
		Control, mean (SD)	*P* value		Intervention, mean (SD)		*P* value		Mean (95% CI)			*P* value		Cohen *d*				
**At 6 months**				.92				.02					.10		0.34		142	
	%	0.03 (1.75)			–0.53 (1.50)				–0.56 (–1.23 to 0.11)									
	mmol/mol	0.29 (22.27)			–5.70 (19.89)				–5.99 (–13.56 to 1.58)									
**At 13 months**				.97				.11					.28		0.21		135	
	%	0.01 (2.09)			–0.41 (1.89)				–0.42 (–1.18 to 0.34)									
	mmol/mol	0.06 (27.31)			–4.44 (24.52)				–4.50 (–12.94 to 2.38)									
**At 18 months**				.63				.002					.09		0.32		121	
	%	–0.14 (2.12)			–0.83 (2.07)				–0.68 (–1.48 to 0.12)									
	mmol/mol	–2.24 (28.31)			–9.01 (23.37)				–6.77 (–15.91 to 2.38)									

^a^Tests of between-group differences used group×time planned contrasts at 6 months, 13 months, and 18 months. Statistical significance for the planned contrasts was defined as 2-sided *P*<.05.

**Figure 4:**
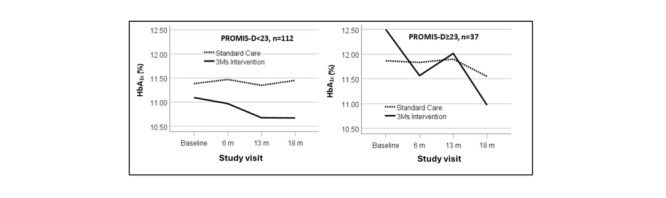
Hemoglobin A_1c_ (HbA_1c_) trajectories by the Patient-Reported Outcome Measurement Information System Pediatric Short Form Depressive Symptoms (PROMIS-D): depressive symptoms low to moderate (<23) versus high (≥23). The high cut-point was 1 SD above the PROMIS-D scale mean at baseline. In the high depressive symptom subgroup, the drops in HbA_1c_ from baseline to 6 months and from baseline to 18 months were significant (*P*<.05).

Examination of tests of post hoc simple effects of PROMIS-D suggested a moderation effect, with the most prominent decreases in HbA_1c_ levels found in the high depressive symptom subgroup whose caregiver received The 3Ms (Figure 4). The effects were significant in the high depression subgroup at 6-month follow-up (decrease of 0.94%, CI –1.68 to –0.19; or 10.25 mmol/mol, CI –18.36 to –2.14; *P*=.01) and 18-month follow-up (decrease of 1.42%, CI –2.32 to –0.53; or 15.68 mmol/mol, CI –25.41 to –5.91; *P*=.002).

## Discussion

While a number of studies have tested the efficacy of eHealth interventions for adolescents with T1D [10], evidence that they improve glycemic control is limited. Those few previous studies testing the efficacy of eHealth interventions to improve the diabetes-related health of Black adolescents used small samples and pilot designs. Lack of attention to the needs of Black families and insufficient focus on the development and testing of relevant, culturally tailored interventions contribute to significant health disparities for this population [38]. In recent years, there has also been a growing interest in the use of technology-based behavioral interventions to promote health in communities of color, as they may circumvent some of the barriers faced by such communities in accessing such services [39].

The results of this study did not support a significant improvement in glycemic control for adolescents in The 3Ms group in comparison to controls overall. However, findings from this study showed a significant moderation effect of baseline depression. Adolescents with higher depressive symptoms were most likely to benefit from The 3Ms, as they had the greatest reductions in glycemic control. The mean reduction in HbA_1c_ levels was 1.4% at 18-month follow-up for this group, which is both statistically significant and clinically meaningful. One-fourth of the present sample of Black youth had elevated symptoms of depression, which is consistent with previous studies showing that youth with T1D are at risk for depression, negative affect, and diabetes distress, as well as current guidelines suggesting that youth with T1D should be screened for depression [40]. Depression and negative affect have been linked to suboptimal glycemic control in previous studies [28]. Our results suggest that increasing parent oversight of daily diabetes care was the most effective for this subset of adolescents, where motivational or other factors associated with depressed mood may interfere with youth completing their routine care. Although not directly measured in the study, adolescents may also have perceived increased parental support, empathy, or warmth when parents engaged in daily oversight of their diabetes management, which could have been of increased benefit for those adolescents experiencing more depressive symptoms.

The use of a multicenter design and the recruitment of adolescents from 7 different clinics in 2 major US cities increase confidence in the generalizability of the findings to samples of urban, low-income, Black youth. However, the findings may not be applicable to rural adolescents or to Black youth of higher socioeconomic status. Study limitations also include the clinic-based intervention delivery approach and the use of a recruitment strategy where only families who obtained their diabetes care in a tertiary care setting were approached. Clinic-based delivery was chosen due to the well-established finding of limited engagement with eHealth interventions that rely on the individual’s own motivation to use them [32]. However, future studies could evaluate the efficacy of The 3Ms if the intervention is provided to caregivers through a cellphone app or freely accessible internet site. Future studies could also investigate barriers and facilitators to broader dissemination of the intervention within pediatric diabetes clinic settings, including the potential value of the intervention for providing family-centered care [41] or culturally competent care for Black youth and their families [42].

In summary, this study demonstrates the potential of a brief, culturally tailored, family-based behavioral intervention delivered during diabetes clinic appointments to improve the health of Black adolescents with T1D, particularly those with depressive symptoms. More research is needed to develop effective interventions to improve health equity for this population.

## Abbreviations

FDA: Food and Drug Administration
HbA_1c_: hemoglobin A_1c_
LME: linear mixed effects
PROMIS-D: Patient-Reported Outcome Measurement Information System Pediatric Short Form Depressive Symptoms
T1D: type 1 diabetes
